# Metabolomic Profiling of the Nectars of *Aquilegia pubescens* and *A*. *Canadensis*

**DOI:** 10.1371/journal.pone.0124501

**Published:** 2015-05-01

**Authors:** Christos Noutsos, Ann M. Perera, Basil J. Nikolau, Samuel M. D. Seaver, Doreen H. Ware

**Affiliations:** 1 Cold Spring Harbor Laboratory, Cold Spring Harbor, New York, United States of America; 2 W.M. Keck Metabolomics Research Laboratory, Iowa State University, Ames, Iowa, United States of America; 3 Department of Biochemistry, Biophysics, and Molecular Biology, Iowa, State University, Ames, Iowa, United States of America; 4 Mathematics and Computer Science Division, Argonne National Laboratory, Argonne, Illinois, United States of America; 5 Computation Institute, The University of Chicago, Chicago, Illinois, United States of America; 6 USDA-ARS-NAA, Robert W. Holley Center, Ithaca, New York, United States of America; National Research Council of Italy, ITALY

## Abstract

To date, variation in nectar chemistry of flowering plants has not been studied in detail. Such variation exerts considerable influence on pollinator–plant interactions, as well as on flower traits that play important roles in the selection of a plant for visitation by specific pollinators. Over the past 60 years the *Aquilegia* genus has been used as a key model for speciation studies. In this study, we defined the metabolomic profiles of flower samples of two *Aquilegia* species, *A*. *Canadensis* and *A*. *pubescens*. We identified a total of 75 metabolites that were classified into six main categories: organic acids, fatty acids, amino acids, esters, sugars, and unknowns. The mean abundances of 25 of these metabolites were significantly different between the two species, providing insights into interspecies variation in floral chemistry. Using the PlantSEED biochemistry database, we found that the majority of these metabolites are involved in biosynthetic pathways. Finally, we explored the annotated genome of *A*. *coerulea*, using the PlantSEED pipeline and reconstructed the metabolic network of *Aquilegia*. This network, which contains the metabolic pathways involved in generating the observed chemical variation, is now publicly available from the DOE Systems Biology Knowledge Base (KBase; http://kbase.us).

## Introduction

Nectar plays an essential role in the interaction between animal- and insect-pollinated plants and their pollinators [1]. Plant–pollinator interactions are beneficial to plants, which require pollination in order to successfully reproduce, as well as to pollinators, which derive food from plant visitation. Key factors affecting the frequency and duration of visitation of pollinators to plants include nectar production rate [2] and the chemical composition of nectar, including the types and relative amounts of sugars, amino acids, organic acids, and lipids [3,4]. In particular, nectar sugar composition has been thoroughly studied in relation to pollinator assemblages in angiosperms [5,6]. Although several studies have compared sugar composition between species, only a few reports have compared individuals, populations, or subspecies of the same species [7,8].

**Fig 1:**
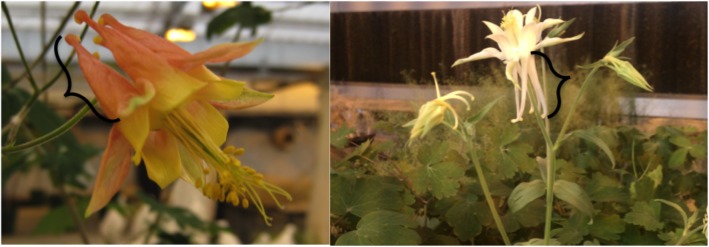
Floral variation. **Black braces denote the spurs, which contain the nectar.** a) *A*. *canadensis;* b) *A*. *pubescens*. The darker-colored region (bottom to middle part) of the spur indicates the nectar level

The *Aquilegia* genus consists of 70 species[9,10]; two of these species, *Aquilegia canadensis* and *Aquilegia pubescens*, exhibit extensive variation in flower morphology [11]. *A*. *canadensis*, which is native to eastern and central North America, has petals that are prolonged backwards into a tubular spur; the petals are primarily yellow, but redden toward the tip of the spur, which is red. Sepals are petal-like. Plants grow from early spring, bloom from March to July, and set fruit in mid-to-late summer. *A*. *pubescens*, which is native to the South Sierra mountains in California, grows only in alpine and subalpine climates; both the sepals and spurs of the flowers are yellow. Flower positioning also differs between *A*. *canadensis* and *A*. *pubescens*: the *A*. *canadensis* flower faces downwards, whereas *A*. *pubescens* flowers are erect rather than drooping. Flower morphology and positioning may be essential determinants of pollinator visitation (Fig 1). Further details regarding these species can be found in [12].

*Aquilegia* species are pollinated by three major categories of pollinators: bumblebees, hummingbirds, and hawk moths. Speciation in genus *Aquilegia* has been proposed to be driven by the pollinator shift model [10]. In this model, nectar spurs evolve increasing length via a directional shift to pollinators with longer tongues. Therefore, the long-spurred *A*. *pubescens* is primarily pollinated by hawk moths, whereas *A*. *canadensis* is pollinated by hummingbirds. Despite the important role played by flower morphology in determining the identity of primary pollinators, nectar composition may also have a larger impact on pollinator attraction, thereby driving diversification. Recently, an elegant study provided evidence that alternations in taste receptor function contributed to the acquisition of nectar-feeding behavior and enabled the extensive radiation of hummingbird species [13].

To identify the key nectar elements that attract different types of pollinators, we sought to characterize the metabolic profiles of *A*. *pubescens* and *A*. *canadensis*. Using computational analysis, we mapped the experimentally identified metabolites to known metabolic pathways in the PlantSEED database. Finally, we extended the reconstruction of metabolic networks of a model species of genus *Aquilegia*, *A*. *coerulea*, to allow researchers to gain insight into the metabolism of a newly sequenced basal eudicot flowering plant.

## Material and Methods

### Plant growth and collection of flowers

*A*. *pubescens* and *A*. *canadensis* plants were grown from seeds in pots in a greenhouse under the following conditions: day temperature, 22°C; night temperature, 19°C; and daylight period, 16 hours. Flowers were monitored as they emerged. To ensure consistency in the comparison of their nectar [14], three male-phased (1 day old),flowers from each species were collected and immediately frozen in liquid nitrogen Flower samples were stored at -80°C prior to metabolic profiling.

### Sample preparation

To isolate the metabolites and for the GC-MS analysis, we followed the methods described by the Metabolomics Standards Initiative[15]. More specifically, the identification of the metabolites was done by comparing the mass spectral to NIST08 Library and using Retention Indices. Because metabolites (known and unknown) respond variably to the MS detector, we quantitated our metabolites relative to internal standards, as described in [16]. Prior to metabolite extraction, ~20 mg of tissue was precisely weighed, spiked with internal standards (20 μg of ribitol and 20 μg of nonadecanoic acid), and homogenized with 0.35 mL of hot methanol (60°C)[17]. The mixture was immediately incubated for 10 min at 60°C and sonicated for 10 min. Chloroform (0.3 mL) and 0.3 mL of water were added, and the mixture was vortexed for 3 min. After centrifugation to separate phases, 0.2 mL of the upper polar phase and 0.2 mL of the lower non-polar phase were removed and transferred into 2-mL glass vials. Both fractions were dried in a SpeedVac concentrator. The extracts were subjected to methoximation with methoxyamine hydrochloride at 30°C for 90 min. Samples were silylated with BSTFA/TCMS at 60°C for 30 min, and then subjected to gas chromatography–mass spectrometry (GC-MS) on an 6890N gas chromatograph in tandem with a 5973MSD detector equipped with a gas ionization detector (gas flow: UHP Helium, 1.0 ml/min.; ionization mode: El; polarity: positive; skimmer/focusing lens voltages: 70 eV). Samples were loaded onto the GC with an 7683B automatic liquid sampler. The mass range was set from 40–1000 *m*/*z*. The separation column was an HP5MSI (30 m long, 0.250 mm ID, 0.25 μm film thickness). All instruments and the column were obtained from Agilent Technologies. The GC-MS protocol is described in in details in [16]. The GC was controlled by the ChemStation software (Agilent)

### Statistical Analysis

Upon analysis and transformation of analyte peaks to numerical values, we implemented filtering of the data. Specifically, values of metabolites detected in three flower samples from a species were selected for further analysis. All statistical analysis was performed on iPlant’s cloud-computing resource, Atmosphere [18], using an image in which R v3.0.1 was installed. For Student’s t-test, we used the built-in *t*.*test* function with the confidence interval set at 0.95. For principal component analysis (PCA), we used the built-in *prcomp* function with the default settings. For visualization of the principal components, we used the R package *ggplot2*. We performed Pearson correlation analysis using the *cor* function.

### Reconstruction of the metabolic network

The *Aquilegia* metabolic network was constructed by annotating the reference genome of *A*. *coerulea* v1.1; sequence data were produced by the US Department of Energy Joint Genome Institute. Specifically, the annotation originated from the PlantSEED database [19], which contains a number of plant protein families curated according to metabolic functions pertaining to plant primary metabolism. Annotation was performed using the RAST server within SEED [20]. All protein sequences in the genome were searched for k-mers of 8 amino acids that were associated with any of the PlantSEED protein families. Once a ‘hit’ was identified, the annotation was propagated to the *Aquilegia* sequence. Once the annotation was complete, a metabolic model was generated from the annotation, using the plant template created by the PlantSEED project. The plant template consists of curated reactions involved in plant primary metabolism and an extensive plant biomass. When the template is used, the annotation in the genome is linked to the reactions in the template, which are then used to create a metabolic reconstruction. Finally, the plant biomass is added to the reconstruction to allow it to function as a model of plant primary metabolism in *Aquilegia*. A gap-filling algorithm was used to find and fill gaps in the reconstruction that would ensure that the metabolic network could generate the same plant biomass using metabolic flux-analysis methods such as flux balance analysis (FBA; see PlantSEED paper for more details). Each step in the generation of the metabolic network was performed within the DOE Systems Biology Knowledgebase environment and framework (KBase; http://kbase.us). The annotated genome and metabolic reconstruction are available for public use in the KBase workspace named “Acoerulea”, which can be accessed by users via a free KBase account (http://narrative.kbase.us/functional-site/#/ws/objects/Acoerulea). The same workspace contains results obtained by simulating heterotrophic growth using flux balance analysis, allowing users to browse reaction activity.

## Results

### Identification of metabolites to *A*. *canadensis* and *A*. *pubescens* species

We performed metabolomic profiling of flowers from three independent plants for each of the two species. We were able to identify a total of 106 analyte peaks, which were integrated with the ChemStation software from Agilent and converted to numerical values based on internal standards, and then filtered. Metabolites were quantitated relative to internal standards, for reasons described in Materials and Methods. Hence, we calculated the standard deviation for the internal standards, highlighted with yellow in S1 Table, for each of the species; all values were very low (nonadecanoic acid: 5.17•10^–5^ for *A*. *canadensis* and 5.14•10^–5^ for *A*. *pubescens*; ribitol: 9.35•10^–5^ for *A*. *canadensis* and 9.29•10^–5^ for *A*. *pubescens*). Using t-test, we ended up with a list of 75 metabolites, including 21 organic acids, 1 fatty acid, 5 amino acids, 4 esters, 25 sugars, and 19 chemically undefined compounds (S1 Table). Of these, 63 were present in both species, 9 were specific to *A*. *pubescens*, and 3 were specific to *A*. *canadensis* (S1 Table). The mean levels of 27% of the metabolites (17 out of 63) metabolites differed significantly (p-value<0.05) between species (Table 1), and 12 additional metabolites were species-specific; thus we identified at total of 29 metabolites that distinguished the two species.

**Table 1:** Metabolites that significantly differ between *A*. *canadensis* and *A*. *pubescens* by Student’s t-test.

	Sample	*A*. *canadensis*				*A*. *pubescens*				pvalue from t-test
		Amount (μmol/mg)				Amount (μmol/mg)				
	Metabolite	1	2	3	Average Amount	1	2	3	Average Amount	
Acid	Propanoic acid, 2,3-bis[oxy]	0.004383	0.00433	0.00391	0.004209	0.00227	0.002435	0.0022	0.0023	0.00
Acid	4-aminobutyric acid	4.73E-06	8.13E-06	1.2E-05	8.41E-06	2.5E-05	3.24E-05	2E-05	2.7E-05	0.01
Acid	Glucaric acid	0.000337	0.000333	0.00028	0.000316	0.00016	0.000165	0.0002	0.00017	0.02
Acid	Tetradecanoic acid	7.86E-05	9.72E-05	5.1E-05	7.57E-05	0	0	0	0	
Acid	Citric acid	0.000332	0.000325	0.00028	0.000312	0	0	0	0	
Acid	2-Isopropylmalic acid	3.97E-05	3.21E-05	3E-05	3.4E-05	0	0	0	0	
Acid	Quinolinic acid	0	0	0	0	0.0004	0.000268	0.0002	0.00029	
aminoacid	Threonine	0.000357	0.000302	0.00029	0.000315	0.00024	0.000256	0.0002	0.00023	0.04
aminoacid	5-oxo-Proline	0.000158	0.000124	0.00011	0.00013	0.00025	0.000275	0.0002	0.00025	0.00
aminoacid	Serine	0	0	0	0	2.6E-05	3.2E-05	3E-05	2.9E-05	
sugar	Ribose	0.001448	0.001492	0.00139	0.001445	0.00115	0.001001	0.001	0.00105	0.00
sugar	1,6-Anhydro-glucose	2.59E-05	2.54E-05	2.1E-05	2.43E-05	4.4E-05	4.88E-05	5E-05	4.6E-05	0.00
sugar	Galactofuranose	0.000139	0.000125	0.00011	0.000124	0.00034	0.000331	0.0003	0.00032	0.00
sugar	Fructose	0.023354	0.023554	0.02285	0.023252	0.02961	0.027627	0.0268	0.02802	0.02
sugar	Glucose	0.014683	0.014752	0.01447	0.014636	0.02067	0.018609	0.0187	0.01934	0.02
sugar	Turanose	1.56E-05	1.52E-05	1.4E-05	1.48E-05	3.7E-05	4.02E-05	4E-05	4E-05	0.00
sugar-alcohol	Glucitol	0.000232	0.000234	0.00021	0.000224	4.1E-05	3.51E-05	3E-05	3.6E-05	0.00
sugar-alcohol	Inositol	0.002304	0.0026	0.00216	0.002356	0.0018	0.001867	0.0016	0.00177	0.03
sugar-alcohol	Glycerol	6.3E-05	7.09E-05	6.7E-05	6.69E-05	4E-05	4.49E-05	4E-05	4.3E-05	0.00
sugar	α-D-Glucopyranoside, β-D-fructofuranosyl- (sucrose)	0.020467	0.017927	0.01401	0.017467	0.00399	0.004812	0.0039	0.00423	0.02
sugar	Xylofuranose	0	0	0	0	2.7E-05	2.41E-05	2E-05	2.3E-05	
sugar	Erythro-Pentofuranose, 2-deoxy	0	0	0	0	0.00017	0.000212	0.0002	0.00019	
ester	Catechollactate	0	0	0	0	5.1E-05	5.97E-05	6E-05	5.7E-05	
ester	Myristic acid, 2,3-bis(oxy)propyl ester	0	0	0	0	1.3E-05	1.45E-05	1E-05	1.3E-05	
sugar	Arabino-Hexos-2-ulose	0	0	0	0	2.6E-05	2.49E-05	1E-05	2.1E-05	
sugar	Xylopyranose	0	0	0	0	2.8E-05	2.09E-05	1E-05	2E-05	
sugar	Xylulose	0	0	0	0	2.6E-05	1.93E-05	1E-05	1.9E-05	
	Unknown 28	5.25E-05	5.95E-05	4.2E-05	5.15E-05	0.00021	0.00022	0.0002	0.00021	0.00
	Unknown 29	0.000137	0.000169	0.00014	0.000148	0.00092	0.000882	0.0008	0.00086	0.00

### Nectar composition

**Fig 2:**
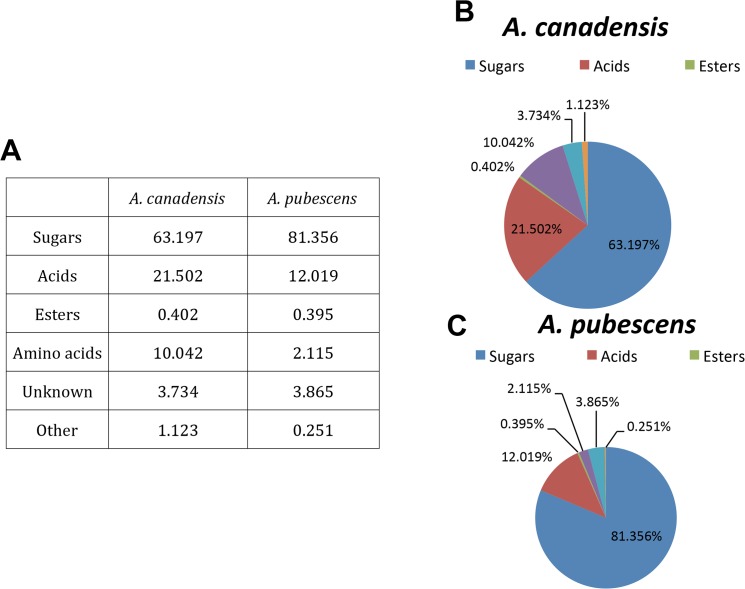
Compositions of compounds in all metabolic categories. A) Percentage of each type of compound in *A*. *canadensis* and *A*. *pubescens*, relative to the total number metabolites. B-C) Pie chart representation of the percentages shown in A.

After identifying metabolites, we examined the two species’ compositions of compounds in all metabolic categories. In the *A*. *canadensis* samples, total metabolites consisted of 63.197% sugars, 21.502% organic acids, 0.402% esters, 10.042% amino acids, and 3.734% unknown compounds and 1.123% other, whereas in *A*. *pubescens*, total metabolites consisted of 81.356% sugars, 12.019% organic acids, 0.395% esters, 2.115% amino acids, and 3.865% unknown compounds and 0.251% other (Fig 2). Thus nectar composition varied significantly between species.

Sugars constituted over 90% of all metabolites that differed significantly between the two species. Of the 15 different sugar metabolites identified, the mean levels of 10 differed significantly between the two species, including the three most abundant: fructose, glucose and sucrose. Notably, fructose and glucose were more abundant than sucrose in *A*. *pubescens*, whereas the sucrose concentration was as high as those of fructose and glucose in *A*. *canadensis*. At the species level, *A*. *canadensis* exhibited slightly higher net total sugars and total metabolites than *A*. *pubescens*. Additionally, we found five sugars (D-xylofuranose, arabino-hexos-2-ulose, α-D-xylopyranose, xylulose, and 2-deoxy-D-erythro-pentofuranose) that were present only in *A*. *canadensis*, suggesting that the two species exhibit qualitative as well as quantitative differences in sugar metabolites.

The second major category of metabolites we detected was organic acids. Of the 21 organic acids we identified, the levels of only three were significantly different between the two species (Table 1). Species-specific organic acids were also detected; citric acid, tetradecanoic acid (fatty acid), and 2-isopropylmalic acid were only detectable in *A*. *canadensis*, whereas quinolinic acid was only detectable in *A*. *pubescens*. The qualitative and quantitative variation in organic acids, in addition to that of sugars, suggests that nectar composition is a complex trait.

Amino acids were also detected as metabolites in nectar. The mean levels of two amino acids (L-threonine, 5-oxo-proline) differed significantly between the two species, while a third amino acid, serine, was present only in *A*. *pubescens*. Glycine and cytosine levels did not differ significantly. In addition to amino acids, we also found esters; two out of four esters were present only in *A*. *pubescens*. Moreover, a sizable fraction of 17 unknown metabolites was detected, only two of which (Unknown #28 and Unknown #29) differed significantly between species; these compounds remain to be identified. The variation in metabolite identities and concentrations prompted us to look for correlations between flower spur length and the levels of metabolites that differed significantly between the two species. With some exceptions, metabolites tended to be positively correlated with spur length in one species and negatively correlated in the other, (Table 2). The most striking exception was the strong positive correlations in *A*. *pubescens* and the strong negative correlations in *A*. *canadensis*, of the amino acids 5-oxo-proline, and L-threonine. Of all sugars, sucrose, scyllo-inositol and β-D- galactofuranose exhibited the highest correlations with spur length, whereas fructose and glucose exhibited very low positive correlations to spur length in both species. Unknown metabolite #29 was among the compounds most strongly positively correlated with spur length in both species. Moreover, at the species level, *A*. *pubescens* consistently exhibited very strong positive correlations of spur length with metabolites that differed significantly between the two species,

**Table 2:** Pearson correlations (r) of spur length with levels of metabolites that differed significantly between *A*. *canadensis* and *A*. *pubescens*.

Metabolite	*A*. *canadensis*	*A*. *pubescens*
Propanoic acid, 2,3-bis[oxy]	-0.28	0.95
4-aminobutyric acid	0.60	0.94
Glucaric acid	-0.024	-0.76
Threonine	-0.85	0.98
5-oxo-Proline, 5-oxo	-0.80	0.99
Ribose	0.26	0.12
1,6-Anhydro-β-d-glycose	-0.28	0.41
Fructose	0.09	0.40
Glycose	0.05	0.06
Turanose	-0.40	-0.54
Glucitol	-0.1	0.46
Inositol	0.51	0.99
Glycerol	0.97	0.03
Sucrose	-0.55	0.86
β-D-Galactofuranose	-0.60	0.83
Unknown 28	0.22	0.98
Unknown 29	0.80	0.80

**Fig 3:**
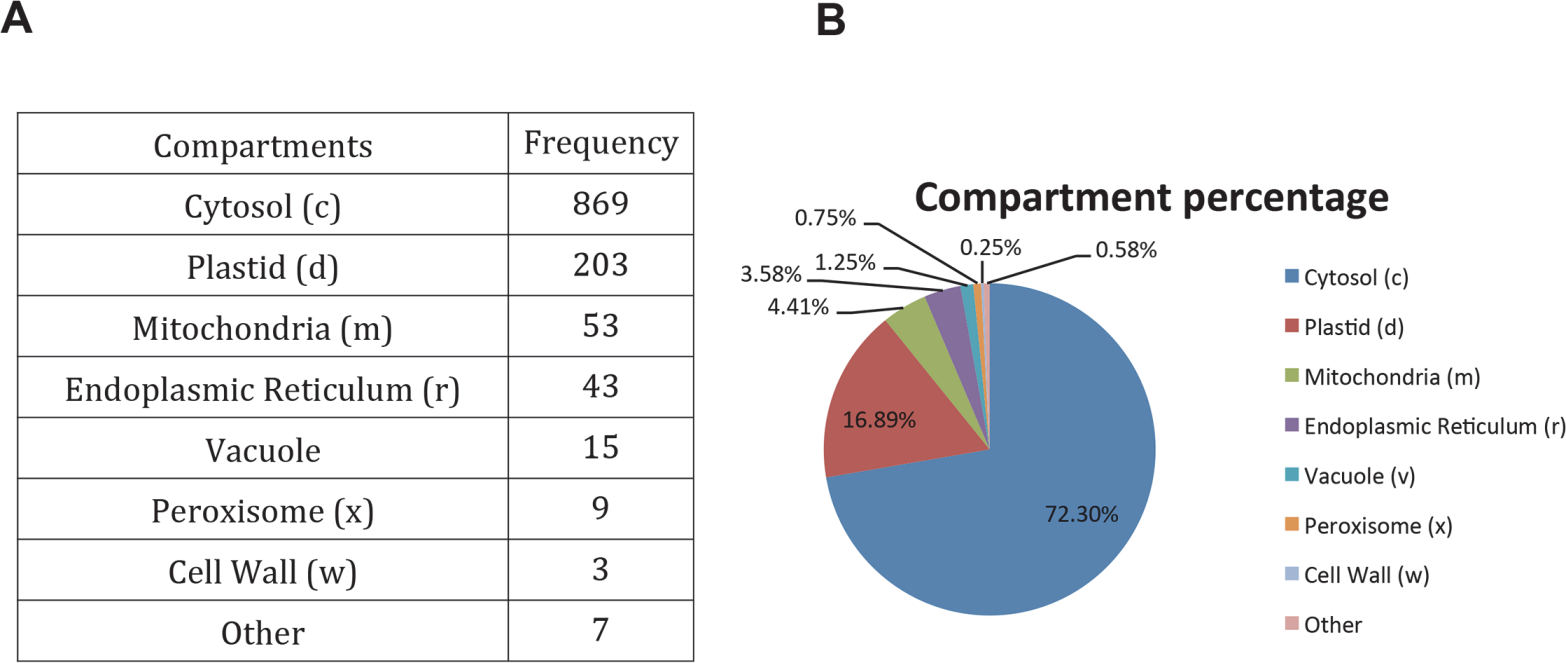
A) A biplot of principal components 1 and 2. The black numbers inside the plot represent each of the metabolites (for a key, see S2 Table) and the red arrow shows the relative loadings of the species to the first and second principal components. B) Is a zoom version of A for a better visualization of the metabolites.

In addition, we performed principal component analysis(PCA) to define the metabolites that contributed the most to differentiation between the two species (Fig 3; Table 3 and S2 Table). The PCA revealed that PC1 explained 90% of the total variance, whereas PC2 explained another 8% (Table 3).

**Table 3:** Summary of principal component analysis of all metabolites that differed significantly between the two species.

	PC1	PC2	PC3	PC4	PC5	PC6
Standard Deviation	2.33	0.67	0.30	1.79	1.23	0.00
Proportion of Variance	0.90	0.08	0.015	0.00	0.00	0.00
Cumulative Proportion	0.90	0.98	0.99	0.99	0.99	1.00

### Mapping of metabolites to pathways and reconstruction of the metabolic network of *Aquilegia*

We mapped all of the identified metabolites to known pathways in the PlantSEED database. Of the 75 metabolites, we were able to map 21. Each metabolite mapped to between 4 and 281 pathways (S3 Table). Because the metabolites are involved in many different pathways, those pathways might be elaborated.

**Fig 4:**
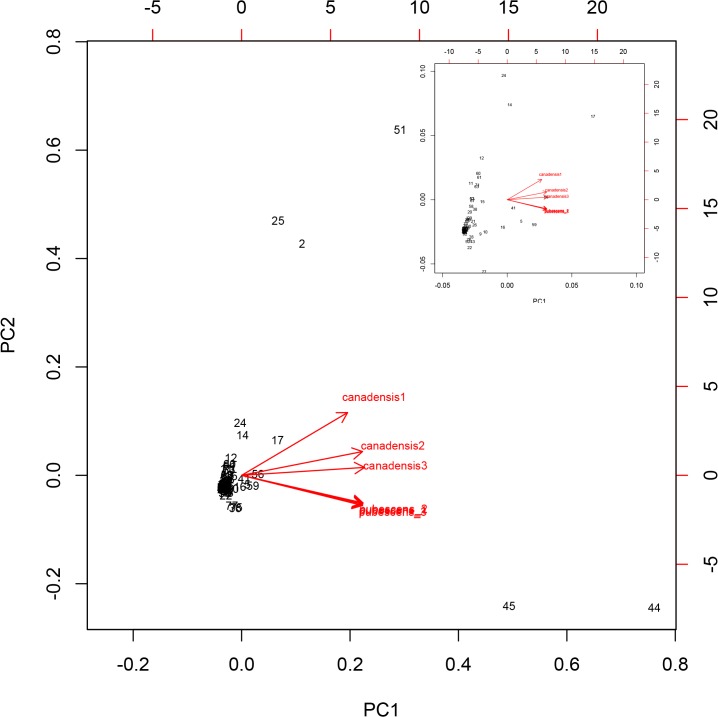
Compartment assignment of metabolic reactions. A) Number of metabolic reactions in each subcellular compartment. B) Pie chart representation of the distribution of metabolic reactions in each compartment, expressed as percentage. Families with fewer than three members were assigned to the ‘Other’ category.

The process of annotating the *Aquilegia* genome resulted in assignment of metabolic annotations to 1,202 different features, and the resulting working metabolic model consisted of 1,573 reactions (S4 Table). The majority of these reactions (72.3%) were assigned to the cytosol, 16.89% to plastids, 4.41% to mitochondria, 3.58% to the endoplasmic reticulum, 1.25% to the cell wall, 0.75% to the vacuole, and 0.25% to peroxisomes and 0.58% to other compartments (Fig 4; S4 Table). According to KEGG pathway annotations (S5 Table), many gene models are involved in metabolism, with the greatest number associated with amino-acid metabolism, fatty acid biosynthesis, and phenylpropanoid biosynthesis, followed by carbon fixation. The whole model is available through KBASE at http://narrative.kbase.us/functional-site/#/ws/objects/Acoerulea.

## Discussion

In this study, by performing metabolomic profiling of whole flowers from two *Aquilegia* species, we sought to obtain insights into the metabolites that influence attraction of pollinators. Some metabolites were present in both species, whereas others were species-specific. Of the shared metabolites, the levels of around 25% differed significantly between the two species. Moreover, we mapped the identified metabolites on to metabolic pathways in the PlantSEED database[19], and constructed a model metabolic network of *A*. *coerulea*.

Sugars are the most prominent component of total metabolites in both species, as described elsewhere [21–23]. Our results revealed that the levels of ten different sugar metabolites differed significantly between the two species we examined. Although the total sugar concentration did not differ significantly between the two species, subtle differences were detected in glucose and fructose levels, as previously reported for species with similar pollinators [2,3,5,24]. Of the three major sugar metabolites, the most pronounced difference found in this study was in the level of sucrose: sucrose levels in *A*. *canadensis*, a hummingbird-pollinated species, was 3.75 times higher than *A*. *pubescens*, a moth-pollinated species. These results are consistent with the sucrose levels detected in naturally grown plants that are visited primarily by hummingbirds [25,26]. Whether pollinator-mediated selection has shaped and/or maintained the differences in nectar between *A*. *pubescens* and *A*. *canadensis* remains to be studied.

Several other sugars also differed significantly between *A*. *pubescens* and A. *canadensis*. The disaccharide turanose, which has a high energy density, is also found in the nectar of sunflowers [27]. Glucitol, also known as sorbitol, is found in traces in the Rosaceae species and seems to be rapidly converted into sucrose and fructose [28]. The levels of scyllo-inositol and glycerol also differed significantly between species. Xylopyranose, a sugar found in the cell walls of plant cells [29], has been previously reported to exhibit species-specific differences in its levels [30], a finding supported by this study.

The results of this study provide insights not only into sugars, which are certainly major metabolites, but also into other types of compounds such as amino acids, organic acids, and esters, which have been neglected in previous studies, despite the fact that they may play significant roles in pollination-driven differentiation. To date, the majority of studies on nectar composition have focused on sugar content [14], even though other metabolites have been demonstrated to be crucial for the attraction of pollinators [31–33].

Amino acids play an important role in the taste of nectar [34,35]. Although the mean total amino-acid content of *A*. *pubescens* was 2-fold greater than that of *A*. *canadensis*, this difference was not statistically significant statistically, as previously reported [36]. However, of the five amino acids we detected, the individual levels of two amino acids (5-oxo-proline and threonine) differed significantly between the two species, and serine was detected only in *A*. *pubescens*. A study that tested the preference of forager honeybees for proline-, serine-, and alanine-enriched nectars reported that proline-enriched nectar was clearly preferred over nectars containing only sugars, whereas serine-enriched nectar was disfavored [37]. Whether similar trends exist for hummingbirds or hawk moths remains to be elucidated.

Organic acids in nectars have not been studied in detail, despite the fact that they may play an important role in nectar quality. For example, studies of honey have revealed that organic acids serve as antioxidants and play a role in preventing infestation [38]. In addition, organic acids provide flavor to nectars, as well as aromas that can attract pollinators. In our study, of the 22 organic acids we identified, three were present in both species at significantly different levels: propanoic acid, 2,3-bis[oxy],4-aminobutyric acid, glucaric acid. Citric acid, 2-isopropylmalic acid, and tetradecanoic acid were unique to *A*. *canadensis*, and quinolinic acid was unique to *A*. *pubescens*. Notably, the proportion of organic acids in nectar was 2-fold greater in *A*. *canadensis* than in *A*. *pubescens*.

Esters tend to provide odor, which along with visual stimuli such as flower color is a vital means of attracting pollinators [39]. Two esters, myristic acid-2,3-bis(oxy)propylester and catechollactate, also differed significantly between the two species, as did the levels of two unknown metabolites (S1 Table). The identities and roles of these compounds should be further explored in future studies.

Early studies [10] elegantly demonstrated the co-evolution of spur length with pollinators in the *Aquilegia* genus. Based on these previous findings, we also examined the correlations between the lengths of the spurs, which contain the nectar, with the levels of various metabolites. Some metabolites were correlated positively with spur length in one species and negatively in the other. *A*. *pubescens* exhibited stronger correlations of spur length with amino acids, acids, and sugars than *A*. *canadensis*, suggesting that various metabolite pathways may be involved in the development of longer spurs. These metabolites should be further tested to provide insights in *Aquilegia* diversification.

Furthermore, we used the PlantSEED database [19] to map experimentally identified metabolites onto metabolic pathways. Only 21 out of our 75 metabolites were mapped to pathways related to biosynthesis. Also, using the PlantSEED pipeline, we annotated the genome of the model species *A*. *coerulea*, whose sequence data are available from JGI, and constructed a metabolic network of the *Aquilegia* in KBase. This metabolic model will enable more *in silico* research on the differential regulation of the enzymes involved in the biosynthesis of the metabolites described in this study.

Variation in nectar production variation among populations, plants, and flowers of a single plant has been observed in multiple species [40]. Moreover, in some cases, nectar volumes and concentrations are strongly affected by environmental factors [41]. Notably, a previous study [14] in *Aquilegia* species pollinated by bees revealed major differences in sucrose and fructose content, not only between the species studied, but also between samples of the same species collected in the field. Because *Aquilegia* species intercross to produce vital hybrids, it would be possible to identify the inheritance of nectar qualitative and quantitative traits by generating crosses between *A*. *canadensis* and *A*. *pubescens* and testing them in a controlled environment.

## Supporting Information

S1 Table Quantification of all identified metabolites. All metabolites and their relative quantities to the internal standards. In the last column are denoted the metabolites different significantly between the two species by performing the t-test
(XLSX)

S2 Table Loadings of Principal Components. The loading are reported for each of the metabolites. In column two there is an ID number for each of the metabolites shown in Fig 4 of the paper.
(XLSX)

S3 Table Metabolites mapped to BioCyc and KEGG Pathways. Metabolites vary to the number of pathways they belong to. We mapped 22 out of our 79 metabolites
(XLSX)

S4 Table Mapping of metabolites to cell compartments and annotations of metabolites. In column F we mention an Arabidopsis exemplar that belongs to each of the pathways. Most of the metabolites belong to cytosol some to the rest of the compartments.
(XLSX)

S5 Table Metabolite percentage involved in the KEGG Pathways. There is large variation on the number of KEGG pathways each of our identified metabolites belongs to.
(XLSX)
