# Correction: Metabolomic Profiling of the Nectars of *Aquilegia pubescens* and *A*. *Canadensis*

**DOI:** 10.1371/journal.pone.0141384

**Published:** 2015-10-22

**Authors:** Christos Noutsos, Ann M. Perera, Basil J. Nikolau, Samuel M. D. Seaver, Doreen H. Ware

There is an error in the title. The correct title is: Metabolomic Profiling of the Nectars of *Aquilegia pubescens* and *A*. *canadensis*. The correct citation is: Noutsos C, Perera AM, Nikolau BJ, Seaver SMD, Ware DH (2015) Metabolomic Profiling of the Nectars of *Aquilegia pubescens* and *A*. *canadensis*. PLoS ONE 10(5): e0124501. doi:10.1371/journal.pone.0124501

The images for Figs 3 and 4 are incorrectly switched. The image that appears as Fig 3 should be Fig 4, and the image that appears as Fig 4 should be Fig 3. The figure captions appear in the correct order. Please see the corrected Fig 3 here.

**Fig 3:**
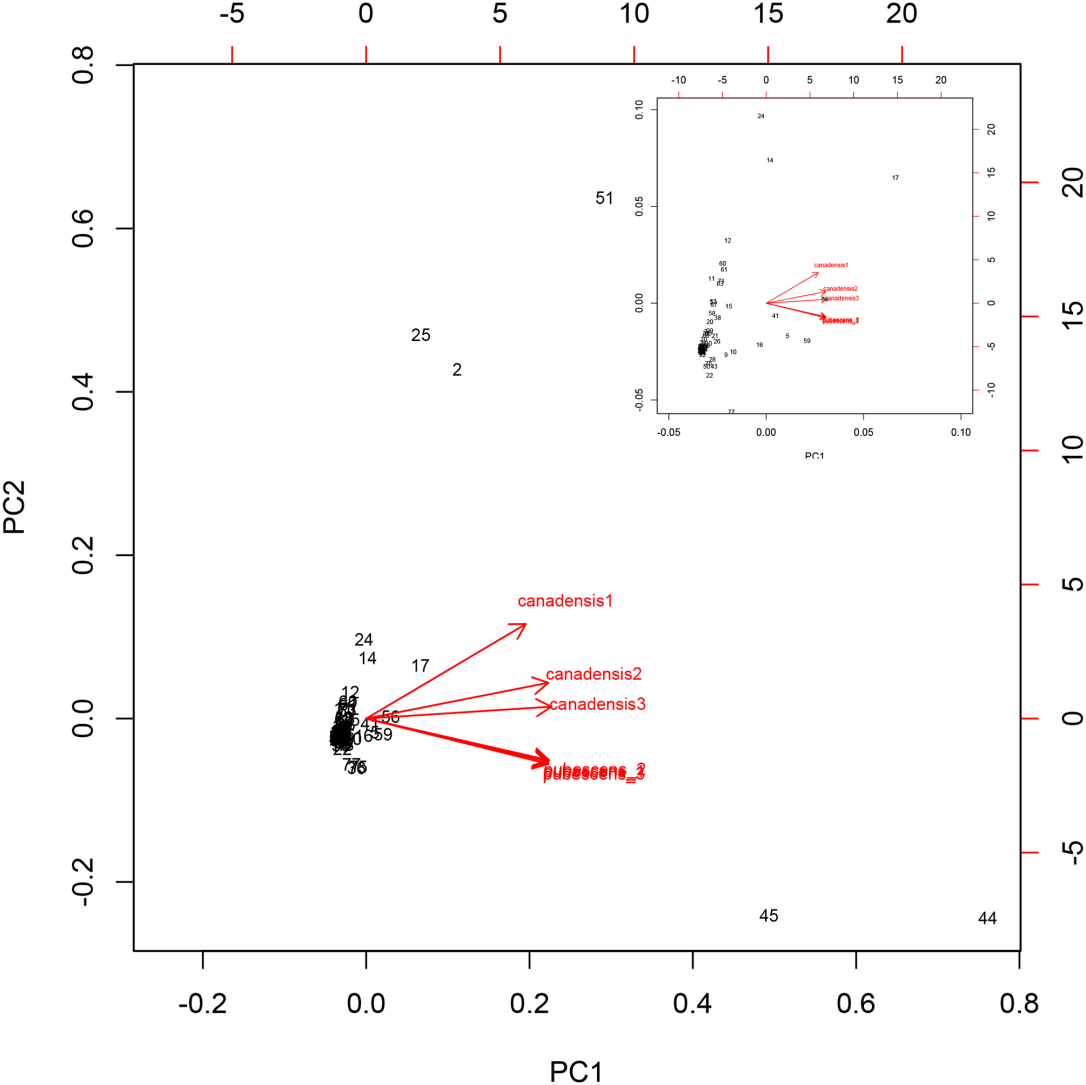
A) A biplot of principal components 1 and 2. The black numbers inside the plot represent each of the metabolites (for a key, see S2 Table) and the red arrow shows the relative loadings of the species to the first and second principal components. B) Is a zoom version of A for a better visualization of the metabolites.

Please see the corrected Fig 4 here.

**Fig 4:**
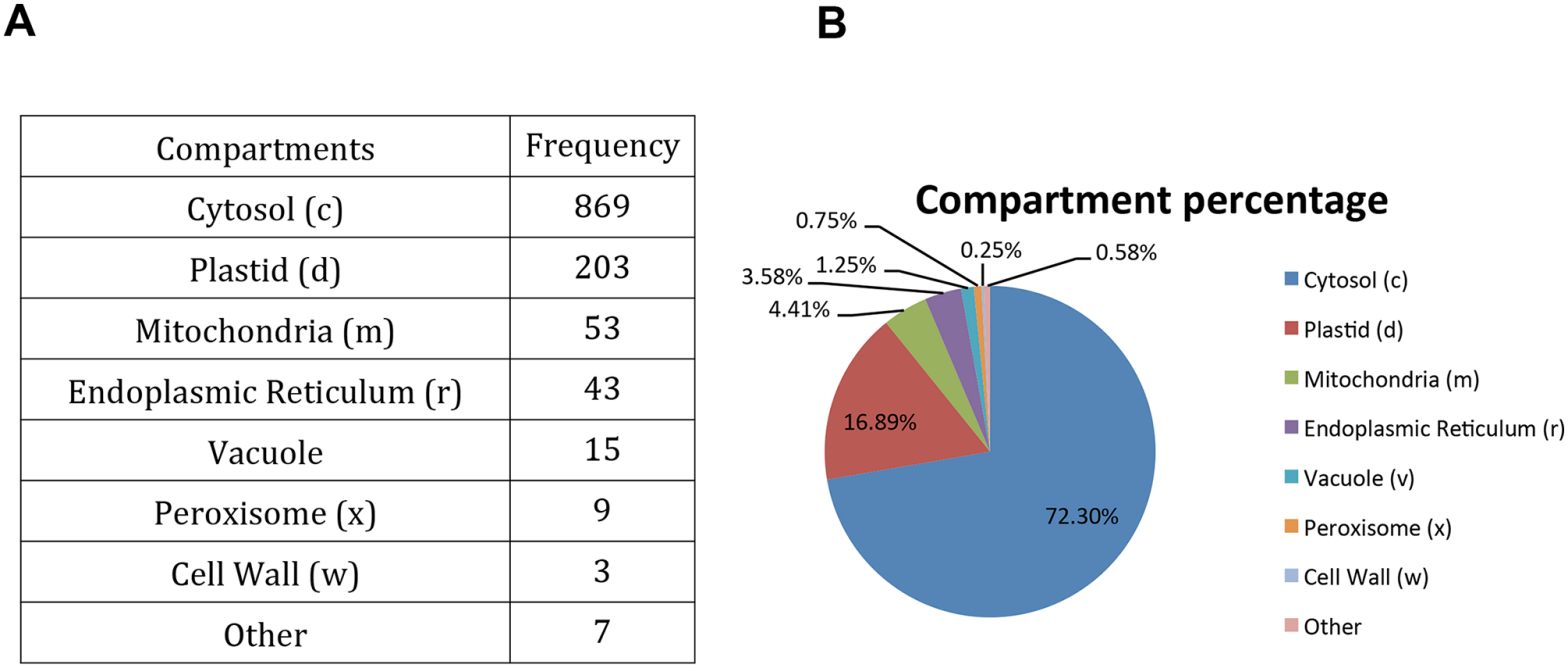
Compartment assignment of metabolic reactions. A) Number of metabolic reactions in each subcellular compartment. B) Pie chart representation of the distribution of metabolic reactions in each compartment, expressed as percentage. Families with fewer than three members were assigned to the ‘Other’ category.
